# Early Life Nutrition, Epigenetics and Programming of Later Life Disease

**DOI:** 10.3390/nu6062165

**Published:** 2014-06-02

**Authors:** Mark H. Vickers

**Affiliations:** Liggins Institute and Gravida, National Centre for Growth and Development, University of Auckland, 85 Park Road, Grafton, Auckland 1142, New Zealand; E-Mail: m.vickers@auckland.ac.nz; Tel.: +64-9-923-6687; Fax +64-9-373-7039

**Keywords:** developmental programming, maternal nutrition, epigenetics, DNA methylation, transgenerational

## Abstract

The global pandemic of obesity and type 2 diabetes is often causally linked to marked changes in diet and lifestyle; namely marked increases in dietary intakes of high energy diets and concomitant reductions in physical activity levels. However, less attention has been paid to the role of developmental plasticity and alterations in phenotypic outcomes resulting from altered environmental conditions during the early life period. Human and experimental animal studies have highlighted the link between alterations in the early life environment and increased risk of obesity and metabolic disorders in later life. This link is conceptualised as the developmental programming hypothesis whereby environmental influences during critical periods of developmental plasticity can elicit lifelong effects on the health and well-being of the offspring. In particular, the nutritional environment in which the fetus or infant develops influences the risk of metabolic disorders in offspring. The late onset of such diseases in response to earlier transient experiences has led to the suggestion that developmental programming may have an epigenetic component, as epigenetic marks such as DNA methylation or histone tail modifications could provide a persistent memory of earlier nutritional states. Moreover, evidence exists, at least from animal models, that such epigenetic programming should be viewed as a transgenerational phenomenon. However, the mechanisms by which early environmental insults can have long-term effects on offspring are relatively unclear. Thus far, these mechanisms include permanent structural changes to the organ caused by suboptimal levels of an important factor during a critical developmental period, changes in gene expression caused by epigenetic modifications (including DNA methylation, histone modification, and microRNA) and permanent changes in cellular ageing. A better understanding of the epigenetic basis of developmental programming and how these effects may be transmitted across generations is essential for the implementation of initiatives aimed at curbing the current obesity and diabetes crisis.

## 1. Background

Obesity and its metabolic sequelae may prove to be the greatest threat to human lifestyle and health in the developed world this century. The obesity epidemic has seen the incidence of being overweight almost double in Western societies in the past two decades and the trend is mirrored in developing nations that are transitioning to first-world economies. Metabolic disease results from a complex interaction of many factors, including genetic, physiologic, behavioural, and environmental influences. The rates at which these metabolic disorders have increased over recent time suggests that environmental (e.g., epigenetic) and behavioural effects, rather than genetic causes, are underpinning the present epidemic.

The Developmental Origins of Health and Disease (DOHaD) hypothesis has highlighted the link between the periconceptual, fetal, and early infant phases of life and the subsequent development of adult obesity and related metabolic disorders. The DOHaD model speculates that the fetus makes predictive adaptations in response to cues from the intrauterine environment, resulting in permanent adjustments in homeostatic systems to aid immediate survival and improve success in an adverse postnatal environment. However, inappropriate interpretations of preenatal cues or changes to that immediate environment may result in a mismatch between prenatal predictions and postnatal reality. As a result, these adaptations, known as predictive adaptive responses (PARS) [1], may ultimately be disadvantageous in postnatal life, leading to an increased risk of chronic diseases in adulthood and/or the inheritance of risk factors and a cycle of disease transmission across generations. In this context, it now well established that alterations in early life nutrition lead to an increased risk for a range of metabolic and cardiovascular disorders in later life [2].

A large number of studies are now focusing on the epigenetic contributions to disease manifestation arising as a consequence of developmental programming. As epigenetic regulation during development undergoes dynamic changes, the epigenome displays a labile nature, which allows it to respond and adapt to environmental stressors, including nutritional modification [3]. As an example, supplementation or restriction of the maternal diet with a range of dietary factors, such as folate, methionine, or choline, has been shown to affect the establishment of DNA methylation patterns in offspring [4,5]. One class of genes that has been considered a potential target or mediator of developmental programming events is imprinted genes, because these genes critically depend upon epigenetic modifications for correct expression and because many imprinted genes have roles in controlling fetal growth as well as neonatal and adult metabolism. As an example, one of the most characterised epigenetically regulated loci, the paternally imprinted insulin-like growth factor-2 (IGF2) gene, is characterised by a labile methylation pattern dependent on the nutritional stimuli received by the growing organism during early life development [6,7]. Of note, work by Waterland showed in post-weaning animals that the paternal allele of IGF2 DMR2 was hypermethylated in the kidneys of mice fed a control synthetic diet. This indicates that the type of post-weaning diet can permanently affect expression of IGF2, suggesting that childhood diet could contribute to IGF2 loss of imprinting in humans [7].

Work in experimental models of programming has now shown that a range of challenges during pregnancy or early neonatal life results in changes in promoter methylation and thus directly or indirectly affect gene expression in pathways associated with a range of physiologic processes [8,9,10,11,12]. For example, epigenetic changes have been observed in p53 in the kidney where uteroplacental insufficiency induces a relative hypomethylation from exon 5 to exon 8, which was associated with deceased mRNA levels of DNMT1 [9]. Epigenetic changes in the maternal low protein (LP) rat model have been reported for the angiotensin II type 1b receptor in the adrenal gland with the proximal promoter of the AT(1b) gene significantly hypomethylated, influencing renal apoptosis and pressor responses [10]. Work by Weaver *et al.* [11] has shown that variations in maternal care directly alter the methylation status of the exon 1_7_ promoter of the GR gene, directly influencing stress responsiveness.

Although most data to data has focused on DNA methylation changes, there are data emerging on histone modifications in the setting of developmental programming. Histone methylation is a key structural alteration that can impact gene expression, either promoting or repressing gene expression, depending on the location. Methylation of either DNA or histone proteins requires methyl donors from dietary folate and requires the presence of vitamins B6 and B12, choline, methionine, and a range of methyltransferases [13,14]. Epigenetics also integrates microRNAs (miRNAs) and there is an emerging framework integrating genomic methylation, histone modifications and the effects of miRNAs. As above, although research to date has predominantly focused on dietary related changes in DNA methylation status [15], an increasing number of reports are highlighting the role of nutrition manipulations on histone structure and function and miRNAs. Work on microRNAs has revealed a complex network of reciprocal interconnections: not only are they able to control gene expression at a post-transcriptional level, thus representing a new important class of regulatory molecules, but they are also directly connected to the epigenetic machinery through a regulatory loop [16]. On one side, the expression of certain miRNAs is controlled by DNA methylation and chromatin modifications. In turn, miRNAs could affect the methylation machinery and the expression of proteins involved in histone modifications. As a combinatorial approach, these mechanisms may then determine gene expression and the resultant phenotype. As an example epigenetic silencing of miRNAs and miRNAs targeting histone deacetylases have been described to play a role in cancer but their roles in the context of nutritional manipulations are not well described.

Of note, the phenotypic effects of epigenetic modifications during development may not manifest until later in life, especially if they affect genes modulating responses to later environmental challenges, such as post-weaning dietary challenges with energy dense diets. The extent of the developmental window for the induction of epigenetic changes in key physiologic systems is not well characterised, but he period of plasticity appears to extend from the periconceptional period into postnatal life [4,11].

## 2. Evidence from Epidemiology

At a mechanistic level, studies in humans linking epigenetic change to metabolic disease risk remain limited although there is evidence for inheritance of tissue specific DNA methylation patterns [17]. Differences in environmental exposure lead to different patterns of epigenetic marking in the somatic tissues of individuals, as evidenced by studies in twins in which DNA methylation and histone acetylation patterns diverged more strongly in older twin pairs with more marked life history differences [18]. Work on human disorders, such as Prader-Willi syndrome (arising due to the absence of the paternally contributed region on chromosome 15q11-q13) or Angelman (lack of maternally-contributed region 15q11) imprinting syndromes, suggested that epigenetic inheritance may play a role in human disorders [19]. Most evidence to date regarding the epigenetic basis of developmental programming has been derived from experimental models and very little human data are available. The Dutch Famine (1944–1945) cohort is one of the most cited cohorts used to examine the effects of poor early life nutrition in humans. Individuals who were prenatally exposed to famine had, six decades later, less DNA methylation of the imprinted IGF2 gene compared with their unexposed, same-sex siblings. The association was specific for periconceptional exposure, reinforcing that very early mammalian development is a crucial period for establishing and maintaining epigenetic marks [20,21].

Although the role of macronutrients is clearly implicated in developmental programming [22], maternal micronutrient levels are of interest as they are essential for the one-carbon metabolism involved in DNA methylation and an imbalance in these nutrients can influence DNA methylation patterns in offspring. As an example, increased maternal vitamin B12 levels during pregnancy are associated with decreased global DNA methylation in newborns while increased serum B12 levels in newborns are associated with reduced methylation of the IGFBP3 gene, involved in intrauterine growth [23]. More recent work in the setting of parental obesity has shown that newborns of obese parents have altered DNA methylation patterns at imprinted genes [24] and paternal obesity has been shown to be associated with IGF2 hypomethylation in newborns [25]. Of note, the significant and independent association between paternal obesity and the offspringʼs methylation status suggests the susceptibility of the developing sperm for environmental insults. The acquired imprint instability may be carried onto the next generation and increase the risk for chronic diseases in adulthood [24].

## 3. Evidence from Animal Models

Several studies, using a range of nutritional challenges, have analysed modifications of DNA methylation patterns to investigate the impact of nutrition on the epigenetic regulation of both imprinted and non-imprinted genes [26]. As described above, alterations in early life nutrition can influence DNA methylation as one-carbon metabolism is dependent upon dietary methyl donors and cofactors including folic acid, choline and vitamin B12 [26,27,28]. Further, maternal undernutrition around the time of conception can induces changes in the expression of miRNAs in offspring which appears to play a role in the development of insulin resistance in later life [29]. Maternal dietary manipulations, such as LP exposure, result in aberrant changes in DNA methylation in key genes changes, which can be prevented by maternal dietary supplementation with cofactors [5,30]. For example, in the rat, altered promoter methylation and gene expression have been shown for the hepatic glucocorticoid receptor (GR) and the peroxisome proliferator-activated receptor α (PPAR-α) [5,31], influencing carbohydrate and lipid metabolism [32]. A maternal LP diet results in hypomethylation of PPAR-α and GR in offspring; alterations which can be normalised to that of controls with maternal folate supplementation [5]. Further, increasing the folic acid content of maternal or post-weaning diets can induce differential changes in phosphoenolpyruvate carboxykinase (PEPCK) mRNA expression and promoter methylation in the rat [33]. In addition to folate, evidence is growing for optimal dietary intake of choline (also involved in one-carbon transfer or methylation) for successful completion of fetal development [34]. Maternal choline supply during pregnancy in the rat modifies fetal histone and DNA methylation, suggesting that a concerted epigenomic mechanism contributes to the long term developmental effects of varied choline intake *in utero* [35]. Choline has been shown to be involved in the methylation of histone H3, expression of histone methyltransferases G9a (Kmt1c) and Suv39h1 (Kmt1a), and DNA methylation of their genes in rat fetal liver and brain [35]. The data on vitamin B12 in animal models are less clear. Vitamin B12 deficiency can result in hypomethylation as, along with folate, B12 is required for the synthesis of methionine and S-adenosyl methionine, the common methyl donor required for the maintenance of methylation patterns in DNA.

Studies in the rodent have primarily utilised maternal undernutrition (either global or low protein) or uterine artery ligation to induce intrauterine growth restricted (IUGR) offspring. In the setting of altered early life nutrition, organisms can fine tune gene expression to achieve environmental adaptation via epigenetic alterations of histone markers of gene accessibility [36]. One example is in experimental models of IUGR where uteroplacental insufficiency leads to decreases in postnatal IGF1 mRNA variants, H3 acetylation and the gene elongation mark histone 3 trimethylation of lysine 36 of the IGF1 gene (H3Me3K36) [36,37]. Further work by Tosh *et al.* [38] also showed that the pattern of early growth following IUGR (rapid *versus* delayed catch-up growth) in the rat leads to differential changes in hepatic IGF1 mRNA expression and histone H3K4 methylation. One of the molecular phenotypes associated with IUGR rats is decreased expression of pancreatic and duodenal homeobox factor-1 (PDX1), a key transcription factor regulating pancreatic development. Recently, reduced PDX1 activity was associated with alterations in histone modifications [39]. Similar findings were observed for the glucose transporter GLUT4 in the muscle of IUGR rats [40]. Some metabolic traits resulting from low birth weights can be transmitted to subsequent generations, suggesting the possibility of epigenetic changes maintained during meiosis. An example of this is the agouti coat-colour variants in mice where coat color variation is correlated to epigenetic marks established early in development [8]. This was evidenced by Dolinoy *et al.* [41] where heterozygous agouti viable yellow mice (A^vy^) were exposed to the flavonoid genistein *in utero* via the maternal diet. This results in an altered coat colour towards pseudo-agouti, and conferred protection against obesity in later life, a hallmark of the agouti phenotype. Work by Waterland *et al.* [42,43] has also utilised mouse models to show that some alleles are particularly susceptible to changes in methylation due to maternal nutrition. This work suggested that maternal nutrition before and during pregnancy may affect the establishment of CpG methylation and the life-long expression of metastable epialleles (epigenetically modified alleles) [43]. In addition to maternal dietary restriction models, a number of reports now suggest that maternal obesity and early life overnutrition can elicit epigenetic changes in offspring. For example, overnutrition during the suckling period can lead to epigenetic modifications in key genes involved in the insulin signaling pathway in skeletal muscle and lead to later development of insulin resistance [44]. Marco *et al.* [45,46] showed that a maternal high fat diet induced hypermethylation of the hypothalamic pro-opiomelanocortin (POMC) promoter and obesity in post-weaning rats and maternal fat intake has been linked to altered epigenetic regulation of genes related to polyunsaturated fatty acid synthesis. Offspring of mothers fed a high fat diet display hepatic cell cycle inhibition and associated changes in gene expression and DNA methylation but these changes did not persist into adulthood [12]. A maternal high fat diet can also alter methylation and gene expression of dopamine and opioid-related genes, thus, is a further potential mechanism for programming of appetite and preference for energy dense foods in postnatal life [47].

In addition to DNA methylation changes, work in primates has shown that a caloric-dense maternal diet leading to obesity can epigenetically alter fetal chromatin structure via covalent modifications of histones [48]. Work by Strakovsky *et al.* [49] showed that a maternal HF diet altered hepatic metabolism in the neonate in a sex-specific manner and these differences, in association with epigenetic modification of histones, may contribute to the known gender differences in oxidative balance. Further, a maternal high fat diet has been reported to modulate fetal surtuin 1 (SIRT1) histone and protein deacetylase activity in nonhuman primates and implicates SIRT1 as a likely mediator of the fetal epigenome and metabolome in the setting of maternal obesity [50]. miRNAs occasionally also cause histone modification and DNA methylation of promoter sites, which affects the expression of target genes [51,52]. Using an ovine model of maternal obesity, fetal muscle miRNA expression is altered and therefore may enhance intramuscular adipogenesis during fetal muscle development [53].

Animal models have also allowed investigation of possible intervention strategies to ameliorate or reverse the effects of developmental programming including those detailed above re maternal methyl donor supplementation. The adipokine leptin has been a recent focus of programming-related studies with neonatal leptin treatment shown to reverse the effects of maternal undernutrition in rodents [54,55]. Mechanisms underlying epigenetic modification of tissue function resulting in a predisposition to altered programming of leptin and insulin signalling are discussed by Holness *et al.* [56]. Leptin has a 3-kb promoter region embedded within a CpG island and contains many putative binding sites for known transcription factors including a glucocorticoid response element. It has been shown that leptin’s promoter is subject to epigenetic programming, and leptin’s expression can be modulated by DNA methylation [57,58,59]. Protective effects of leptin during the suckling period against later obesity may be associated with changes in promoter methylation of the hypothalamic POMC gene [60]. In addition, activation of the leptin receptor also induces expression of suppressor of cytokine signaling-3 (SOCS-3). This protein inhibits further leptin signal transduction and also potently inhibits signalling by the insulin receptor. Altered SOCS-3 methylation may therefore have lasting effects on the leptin-insulin feedback loop (the adipoinsular axis) and adversely impact developmental programming [56]. Yokomori *et al.* [61] have shown that methylation of specific CpG sites and a methylation-sensitive protein could contribute to leptin gene expression during adipocyte differentiation in 3T3-L1 cells. The same laboratory group has also shown that both methylation of specific CpG sites and a methylation-sensitive transcription factor contributes to GLUT4 gene regulation during preadipocyte to adipocyte differentiation [62]. In addition, differential DNA methylation was observed in promoters of genes involved in glucose metabolism including GLUT4 [62] and uncoupling protein 2 (UCP-2) [63]. It addition to leptin, it has been shown that the GLP-1 analog Exendin-4 increases histone acetylase activity and reverses epigenetic modifications that silence PDX1 in the IUGR rat [64]. Further, in line with lifestyle modifications preventing mitochondrial alterations and metabolic disorders, exercise has also been shown to change DNA methylation of the promoter of PGC1a to favor gene expression responsible for mitochondrial biogenesis and function [65]. Dietary supplementation with methyl donors has also been well described. Maternal methylation donor supplementation reduces fatty liver and modifies the fatty acid synthase DNA methylation profile in rats fed an obesogenic diet [66]. Similarly, maternal methyl donor supplementation during lactation can prevent the hyperhomocysteinemia induced by a high-fat-sucrose intake by dams [67].

Aims currently pursued are the early identification of epigenetic biomarkers concerned in individual’s disease susceptibility and the description of protocols for tailored dietary treatments/advice to counterbalance adverse epigenomic events. These approaches will allow diagnosis and prognosis implementation and facilitate therapeutic strategies in a personalised “epigenomically modeled” manner to combat obesity and metabolic disorders [68].

## 4. Transgenerational Epigenetic Programming

The experimental and human evidence to date suggests that developmental programming should be regarded as a transgenerational phenomenon and is therefore often viewed as a form of epigenetic inheritance, either via the maternal or paternal line. Evidence exists for both germline and somatic inheritance of epigenetic modifications which may be responsible for phenotypic changes in further generations [69]. Transgenerational epigenetic transmission of traits allows future generations to be maximally competitive in their environment [70]. Under this assumption, adaptive gene programs acquired during the parental lifespan persist in the subsequent generation, enabling future generations to better survive in a potentially adverse environment. However, evidence suggests that environmental exposures such as poor early life nutrition result in maladaptive parental responses that can be passed to offspring. These epigenetic traits have the potential to result in a population-wide manifestation of a phenotype over several generations—such transmission can exacerbate the rapid onset of phenotypes such as obesity and diabetes currently observed in human populations [70].

A number of nutritional challenges can induce transgenerational, non-genomically determined phenotypic changes in mammals [19]. For example changes in rodents the methylation of the GR promoter in the liver are also reflected in the F2 generation in the absence of any dietary manipulation of F1 female offspring [71]. Work by Waterland *et al.* [72] demonstrated that methyl donor supplementation could prevent transgenerational amplification of obesity. Although a number of studies have now reported transmission to the F2 lineage, transmission to the F3 or subsequent generation (a true marker of transgenerational transmission as it avoids the confounding contributions of the initial maternal insult) is less clear with some studies reporting a resolution of the phenotype by the F3 generation. In the meta-analysis by Aiken and Ozanne, of nine transgenerational studies carried through to F3, five failed to show any effect [69]. The impact of paternal nutritional background in transgenerational inheritance has also been reported in recent work by Fullston *et al.* [73]. Paternal obesity was shown to initiate metabolic disturbances in two generations of mice albeit with incomplete penetrance to the F2 generation. Diet-induced paternal obesity modulated sperm microRNA content and germ methylation status which are potential signals that program offspring health and initiate the transmission of obesity to future generations. Studies in F1 sperm have suggested a role for altered IGF2 and H19 expression in transmission of a phenotype to the F2 offspring [74]. However, not all studies reporting a paternal line transmission have reported epigenetic alterations in the F1 sperm [75]. Work by Radford *et al.* [76] did not show any evidence that the epigenetic reprogramming of imprinting control regions in the germline was susceptible to nutritional restriction thus suggesting that mechanisms other than direct germline transmission are responsible.

Although transgenerational phenotype transmission is often seen as a form of epigenetic inheritance there is also evidence for non-genomic components and the interaction between the developing fetus with the *in utero* environment in perpetuation of programmed phenotypes. These include a suboptimal reproductive tract environment, altered maternal adaptations to pregnancy or other societal factors. Work by Aiken and Ozanne suggests that developmental programming effects could be propagated through the maternal line *de novo* in generations beyond F2 as a consequence of development in a suboptimal intrauterine tract and not necessarily though directly transmitted epigenetic mechanisms. Further, as the effects of age exacerbate the programmed metabolic phenotype, advancing maternal age may increase the likelihood of developmental programming effects being transmitted to future generations [69].

## 5. Conclusions

A wide range of nutritional interventions in pregnancy and lactation, including both undernutrition and maternal obesity, can lead to range of metabolic disorders in offspring, which are mediated in part by epigenetic processes encompassing the chromatin information encrypted by DNA methylation patterns, histone covalent modifications and non-coding RNA or microRNA [68]. Defining the mechanisms underpinning transmission of developmental programming is an area urgently requiring further research and is particularly relevant to populations in transition between traditional and Western lifestyles. That some traits appear to be resolved where others persist suggests that divergent mechanisms of transmission are involved and that those metabolic traits that do persist are capable of being transmitted via the male germline [70]. However, human evidence remains largely unsubstantiated with the strongest argument for transgenerational epigenetic inheritance in humans being data derived from the rodent [77]. Understanding the role of early life nutrition and mechanisms of transgenerational epigenetic inheritance is essential for the development of future intervention strategies to modulate not only that of the immediate adult phenotype but also that of offspring, grandoffspring and beyond. The evidence surrounding maternal folic acid and vitamin supplements, one carbon metabolism and altered DNA methylation patterns also raises the issue that more attention should be given to the potential long-term effects of such supplements on offspring given that around 80% of women in the US take supplements during pregnancy. Since the epigenetic processes are long-term and potentially reversible, once the mechanistic basis of the disease is understood, intervention and strategies aimed at reversal can be devised and implemented. However, there are still many key questions to be answered: How plastic is the system for intervention and what are the critical windows of development at which strategies should be targeted; how many generations does it take to reverse epigenetic imprinting and can reliable markers be developed for disease prediction? [78]

## References

[1] Gluckman P.D., Hanson M.A., Beedle A.S., Spencer H.G. (2008). Predictive adaptive responses in perspective. Trends Endocrinol. Metab..

[2] Fernandez-Twinn D.S., Ozanne S.E. (2010). Early life nutrition and metabolic programming. Ann. N. Y. Acad. Sci..

[3] Jang H., Serra C. (2014). Nutrition, epigenetics, and diseases. Clin. Nutr. Res..

[4] Sinclair K.D., Allegrucci C., Singh R., Gardner D.S., Sebastian S., Bispham J., Thurston A., Huntley J.F., Rees W.D., Maloney C.A. (2007). DNA methylation, insulin resistance, and blood pressure in offspring determined by maternal periconceptional B vitamin and methionine status. Proc. Natl. Acad. Sci. USA.

[5] Lillycrop K.A., Phillips E.S., Jackson A.A., Hanson M.A., Burdge G.C. (2005). Dietary protein restriction of pregnant rats induces and folic acid supplementation prevents epigenetic modification of hepatic gene expression in the offspring. J. Nutr..

[6] Murphy S.K., Jirtle R.L. (2003). Imprinting evolution and the price of silence. Bioessays.

[7] Waterland R.A., Lin J.R., Smith C.A., Jirtle R.L. (2006). Post-weaning diet affects genomic imprinting at the insulin-like growth factor 2 (Igf2) locus. Hum. Mol. Genet..

[8] Jirtle R.L., Skinner M.K. (2007). Environmental epigenomics and disease susceptibility. Nat. Rev. Genet..

[9] Pham T.D., MacLennan N.K., Chiu C.T., Laksana G.S., Hsu J.L., Lane R.H. (2003). Uteroplacental insufficiency increases apoptosis and alters p53 gene methylation in the full-term IUGR rat kidney. Am. J. Physiol. Regul. Integr. Comp. Physiol..

[10] Bogdarina I., Welham S., King P.J., Burns S.P., Clark A.J. (2007). Epigenetic modification of the renin-angiotensin system in the fetal programming of hypertension. Circ. Res..

[11] Weaver I.C., Cervoni N., Champagne F.A., D’Alessio A.C., Sharma S., Seckl J.R., Dymov S., Szyf M., Meaney M.J. (2004). Epigenetic programming by maternal behavior. Nat. Neurosci..

[12] Dudley K.J., Sloboda D.M., Connor K.L., Beltrand J., Vickers M.H. (2011). Offspring of mothers fed a high fat diet display hepatic cell cycle inhibition and associated changes in gene expression and DNA methylation. PLoS One.

[13] Wolffe A.P. (1998). Packaging principle: How DNA methylation and histone acetylation control the transcriptional activity of chromatin. J. Exp. Zool..

[14] Callinan P.A., Feinberg A.P. (2006). The emerging science of epigenomics. Hum. Mol. Genet..

[15] Delage B., Dashwood R.H. (2008). Dietary manipulation of histone structure and function. Annu. Rev. Nutr..

[16] Iorio M.V., Piovan C., Croce C.M. (2010). Interplay between microRNAs and the epigenetic machinery: An intricate network. Biochim. Biophys. Acta.

[17] Silva A.J., White R. (1988). Inheritance of allelic blueprints for methylation patterns. Cell.

[18] Fraga M.F., Ballestar E., Paz M.F., Ropero S., Setien F., Ballestar M.L., Heine-Suner D., Cigudosa J.C., Urioste M., Benitez J. (2005). Epigenetic differences arise during the lifetime of monozygotic twins. Proc. Natl. Acad. Sci. USA.

[19] Kaminsky Z., Wang S.C., Petronis A. (2006). Complex disease, gender and epigenetics. Ann. Med..

[20] Heijmans B.T., Tobi E.W., Stein A.D., Putter H., Blauw G.J., Susser E.S., Slagboom P.E., Lumey L.H. (2008). Persistent epigenetic differences associated with prenatal exposure to famine in humans. Proc. Natl. Acad. Sci. USA.

[21] Tobi E.W., Lumey L.H., Talens R.P., Kremer D., Putter H., Stein A.D., Slagboom P.E., Heijmans B.T. (2009). DNA methylation differences after exposure to prenatal famine are common and timing- and sex-specific. Hum. Mol. Genet..

[22] McMillen I.C., MacLaughlin S.M., Muhlhausler B.S., Gentili S., Duffield J.L., Morrison J.L. (2008). Developmental origins of adult health and disease: The role of periconceptional and foetal nutrition. Basic Clin. Pharmacol. Toxicol..

[23] McKay J.A., Groom A., Potter C., Coneyworth L.J., Ford D., Mathers J.C., Relton C.L. (2012). Genetic and non-genetic influences during pregnancy on infant global and site specific DNA methylation: Role for folate gene variants and vitamin B12. PLoS One.

[24] Soubry A., Murphy S.K., Wang F., Huang Z., Vidal A.C., Fuemmeler B.F., Kurtzberg J., Murtha A., Jirtle R.L., Schildkraut J.M. (2013). Newborns of obese parents have altered DNA methylation patterns at imprinted genes. Int. J. Obes. (Lond.).

[25] Soubry A., Schildkraut J.M., Murtha A., Wang F., Huang Z., Bernal A., Kurtzberg J., Jirtle R.L., Murphy S.K., Hoyo C. (2013). Paternal obesity is associated with IGF2 hypomethylation in newborns: Results from a Newborn Epigenetics Study (NEST) cohort. BMC Med..

[26] Gicquel C., El-Osta A., Le Bouc Y. (2008). Epigenetic regulation and fetal programming. Best Pract. Res. Clin. Endocrinol. Metab..

[27] MacLennan N.K., James S.J., Melnyk S., Piroozi A., Jernigan S., Hsu J.L., Janke S.M., Pham T.D., Lane R.H. (2004). Uteroplacental insufficiency alters DNA methylation, one-carbon metabolism, and histone acetylation in IUGR rats. Physiol. Genomics.

[28] Vanhees K., Vonhogen I.G., van Schooten F.J., Godschalk R.W. (2014). You are what you eat, and so are your children: The impact of micronutrients on the epigenetic programming of offspring. Cell. Mol. Life Sci..

[29] Lie S., Morrison J.L., Williams-Wyss O., Suter C.M., Humphreys D.T., Ozanne S.E., Zhang S., Maclaughlin S.M., Kleemann D.O., Walker S.K. (2014). Periconceptional undernutrition programs changes in insulin-signaling molecules and microRNAs in skeletal muscle in singleton and twin fetal sheep. Biol. Reprod..

[30] Cho C.E., Sanchez-Hernandez D., Reza-Lopez S.A., Huot P.S., Kim Y.I., Anderson G.H. (2013). High folate gestational and post-weaning diets alter hypothalamic feeding pathways by DNA methylation in Wistar rat offspring. Epigenetics.

[31] Lillycrop K.A., Slater-Jefferies J.L., Hanson M.A., Godfrey K.M., Jackson A.A., Burdge G.C. (2007). Induction of altered epigenetic regulation of the hepatic glucocorticoid receptor in the offspring of rats fed a protein-restricted diet during pregnancy suggests that reduced DNA methyltransferase-1 expression is involved in impaired DNA methylation and changes in histone modifications. Br. J. Nutr..

[32] Burdge G.C., Lillycrop K.A., Jackson A.A., Gluckman P.D., Hanson M.A. (2007). The nature of the growth pattern and of the metabolic response to fasting in the rat are dependent upon the dietary protein and folic acid intakes of their pregnant dams and post-weaning fat consumption. Br. J. Nutr..

[33] Hoile S.P., Lillycrop K.A., Grenfell L.R., Hanson M.A., Burdge G.C. (2012). Increasing the folic acid content of maternal or post-weaning diets induces differential changes in phosphoenolpyruvate carboxykinase mRNA expression and promoter methylation in rats. Br. J. Nutr..

[34] Zeisel S.H. (2009). Importance of methyl donors during reproduction. Am. J. Clin. Nutr..

[35] Davison J.M., Mellott T.J., Kovacheva V.P., Blusztajn J.K. (2009). Gestational choline supply regulates methylation of histone H3, expression of histone methyltransferases G9a (Kmt1c) and Suv39h1 (Kmt1a), and DNA methylation of their genes in rat fetal liver and brain. J. Biol. Chem..

[36] Zinkhan E.K., Fu Q., Wang Y., Yu X., Callaway C.W., Segar J.L., Scholz T.D., McKnight R.A., Joss-Moore L., Lane R.H. (2012). Maternal hyperglycemia disrupts histone 3 lysine 36 trimethylation of the IGF-1 gene. J. Nutr. Metab..

[37] Fu Q., McKnight R.A., Yu X., Wang L., Callaway C.W., Lane R.H. (2004). Uteroplacental insufficiency induces site-specific changes in histone H3 covalent modifications and affects DNA-histone H3 positioning in day 0 IUGR rat liver. Physiol. Genomics.

[38] Tosh D.N., Fu Q., Callaway C.W., McKnight R.A., McMillen I.C., Ross M.G., Lane R.H., Desai M. (2010). Epigenetics of programmed obesity: Alteration in IUGR rat hepatic IGF1 mRNA expression and histone structure in rapid *vs.* delayed postnatal catch-up growth. Am. J. Physiol. Gastrointest. Liver Physiol..

[39] Park J.H., Stoffers D.A., Nicholls R.D., Simmons R.A. (2008). Development of type 2 diabetes following intrauterine growth retardation in rats is associated with progressive epigenetic silencing of Pdx1. J. Clin. Investig..

[40] Raychaudhuri N., Raychaudhuri S., Thamotharan M., Devaskar S.U. (2008). Histone code modifications repress glucose transporter 4 expression in the intrauterine growth-restricted offspring. J. Biol. Chem..

[41] Dolinoy D.C., Weidman J.R., Waterland R.A., Jirtle R.L. (2006). Maternal genistein alters coat color and protects Avy mouse offspring from obesity by modifying the fetal epigenome. Environ. Health Perspect..

[42] Waterland R.A., Michels K.B. (2007). Epigenetic epidemiology of the developmental origins hypothesis. Annu. Rev. Nutr..

[43] Waterland R.A., Dolinoy D.C., Lin J.R., Smith C.A., Shi X., Tahiliani K.G. (2006). Maternal methyl supplements increase offspring DNA methylation at Axin Fused. Genesis.

[44] Liu H.W., Mahmood S., Srinivasan M., Smiraglia D.J., Patel M.S. (2013). Developmental programming in skeletal muscle in response to overnourishment in the immediate postnatal life in rats. J. Nutr. Biochem..

[45] Marco A., Kisliouk T., Weller A., Meiri N. (2013). High fat diet induces hypermethylation of the hypothalamic Pomc promoter and obesity in post-weaning rats. Psychoneuroendocrinology.

[46] Hoile S.P., Irvine N.A., Kelsall C.J., Sibbons C., Feunteun A., Collister A., Torrens C., Calder P.C., Hanson M.A., Lillycrop K.A. (2013). Maternal fat intake in rats alters 20, 4*n*-6 and 22, 6*n*-3 status and the epigenetic regulation of Fads2 in offspring liver. J. Nutr. Biochem..

[47] Vucetic Z., Kimmel J., Totoki K., Hollenbeck E., Reyes T.M. (2010). Maternal high-fat diet alters methylation and gene expression of dopamine and opioid-related genes. Endocrinology.

[48] Aagaard-Tillery K.M., Grove K., Bishop J., Ke X., Fu Q., McKnight R., Lane R.H. (2008). Developmental origins of disease and determinants of chromatin structure: Maternal diet modifies the primate fetal epigenome. J. Mol. Endocrinol..

[49] Strakovsky R.S., Zhang X., Zhou D., Pan Y.X. (2014). The regulation of hepatic Pon1 by a maternal high-fat diet is gender specific and may occur through promoter histone modifications in neonatal rats. J. Nutr. Biochem..

[50] Suter M.A., Chen A., Burdine M.S., Choudhury M., Harris R.A., Lane R.H., Friedman J.E., Grove K.L., Tackett A.J., Aagaard K.M. (2012). A maternal high-fat diet modulates fetal SIRT1 histone and protein deacetylase activity in nonhuman primates. FASEB J..

[51] Tan Y., Zhang B., Wu T., Skogerbo G., Zhu X., Guo X., He S., Chen R. (2009). Transcriptional inhibiton of Hoxd4 expression by miRNA-10a in human breast cancer cells. BMC Mol. Biol..

[52] Hawkins P.G., Morris K.V. (2008). RNA and transcriptional modulation of gene expression. Cell Cycle.

[53] Yan X., Huang Y., Zhao J.X., Rogers C.J., Zhu M.J., Ford S.P., Nathanielsz P.W., Du M. (2013). Maternal obesity downregulates microRNA let-7g expression, a possible mechanism for enhanced adipogenesis during ovine fetal skeletal muscle development. Int. J. Obes. (Lond.).

[54] Vickers M.H., Gluckman P.D., Coveny A.H., Hofman P.L., Cutfield W.S., Gertler A., Breier B.H., Harris M. (2005). Neonatal leptin treatment reverses developmental programming. Endocrinology.

[55] Gluckman P.D., Lillycrop K.A., Vickers M.H., Pleasants A.B., Phillips E.S., Beedle A.S., Burdge G.C., Hanson M.A. (2007). Metabolic plasticity during mammalian development is directionally dependent on early nutritional status. Proc. Natl. Acad. Sci. USA.

[56] Holness M.J., Sugden M.C. (2006). Epigenetic regulation of metabolism in children born small for gestational age. Curr. Opin. Clin. Nutr. Metab. Care.

[57] Iliopoulos D., Malizos K.N., Tsezou A. (2007). Epigenetic regulation of leptin affects MMP-13 expression in osteoarthritic chondrocytes: Possible molecular target for osteoarthritis therapeutic intervention. Ann. Rheum. Dis..

[58] Melzner I., Scott V., Dorsch K., Fischer P., Wabitsch M., Bruderlein S., Hasel C., Moller P. (2002). Leptin gene expression in human preadipocytes is switched on by maturation-induced demethylation of distinct CpGs in its proximal promoter. J. Biol. Chem..

[59] Stoger R. (2006). *In vivo* methylation patterns of the leptin promoter in human and mouse. Epigenetics.

[60] Palou M., Pico C., McKay J.A., Sanchez J., Priego T., Mathers J.C., Palou A. (2011). Protective effects of leptin during the suckling period against later obesity may be associated with changes in promoter methylation of the hypothalamic pro-opiomelanocortin gene. Br. J. Nutr..

[61] Yokomori N., Tawata M., Onaya T. (2002). DNA demethylation modulates mouse leptin promoter activity during the differentiation of 3T3-L1 cells. Diabetologia.

[62] Yokomori N., Tawata M., Onaya T. (1999). DNA demethylation during the differentiation of 3T3-L1 cells affects the expression of the mouse GLUT4 gene. Diabetes.

[63] Carretero M.V., Torres L., Latasa U., Garcia-Trevijano E.R., Prieto J., Mato J.M., Avila M.A. (1998). Transformed but not normal hepatocytes express UCP2. FEBS Lett..

[64] Pinney S.E., Jaeckle Santos L.J., Han Y., Stoffers D.A., Simmons R.A. (2011). Exendin-4 increases histone acetylase activity and reverses epigenetic modifications that silence Pdx1 in the intrauterine growth retarded rat. Diabetologia.

[65] Cheng Z., Almeida F.A. (2014). Mitochondrial alteration in type 2 diabetes and obesity: An epigenetic link. Cell Cycle.

[66] Cordero P., Gomez-Uriz A.M., Campion J., Milagro F.I., Martinez J.A. (2013). Dietary supplementation with methyl donors reduces fatty liver and modifies the fatty acid synthase DNA methylation profile in rats fed an obesogenic diet. Genes Nutr..

[67] Cordero P., Milagro F.I., Campion J., Martinez J.A. (2013). Maternal methyl donors supplementation during lactation prevents the hyperhomocysteinemia induced by a high-fat-sucrose intake by dams. Int. J. Mol. Sci..

[68] Martinez J.A., Cordero P., Campion J., Milagro F.I. (2012). Interplay of early-life nutritional programming on obesity, inflammation and epigenetic outcomes. Proc. Nutr. Soc..

[69] Aiken C.E., Ozanne S.E. (2014). Transgenerational developmental programming. Hum. Reprod. Update.

[70] Dunn G.A., Bale T.L. (2011). Maternal high-fat diet effects on third-generation female body size via the paternal lineage. Endocrinology.

[71] Benyshek D.C., Johnston C.S., Martin J.F. (2006). Glucose metabolism is altered in the adequately-nourished grand-offspring (F3 generation) of rats malnourished during gestation and perinatal life. Diabetologia.

[72] Waterland R.A., Travisano M., Tahiliani K.G., Rached M.T., Mirza S. (2008). Methyl donor supplementation prevents transgenerational amplification of obesity. Int. J. Obes. (Lond.).

[73] Fullston T., Ohlsson Teague E.M., Palmer N.O., DeBlasio M.J., Mitchell M., Corbett M., Print C.G., Owens J.A., Lane M. (2013). Paternal obesity initiates metabolic disturbances in two generations of mice with incomplete penetrance to the F2 generation and alters the transcriptional profile of testis and sperm microRNA content. FASEB J..

[74] Ding G.L., Wang F.F., Shu J., Tian S., Jiang Y., Zhang D., Wang N., Luo Q., Zhang Y., Jin F. (2012). Transgenerational glucose intolerance with Igf2/H19 epigenetic alterations in mouse islet induced by intrauterine hyperglycemia. Diabetes.

[75] Drake A.J., Liu L., Kerrigan D., Meehan R.R., Seckl J.R. (2011). Multigenerational programming in the glucocorticoid programmed rat is associated with generation-specific and parent of origin effects. Epigenetics.

[76] Radford E.J., Isganaitis E., Jimenez-Chillaron J., Schroeder J., Molla M., Andrews S., Didier N., Charalambous M., McEwen K., Marazzi G. (2012). An unbiased assessment of the role of imprinted genes in an intergenerational model of developmental programming. PLoS Genet..

[77] Morgan D.K., Whitelaw E. (2008). The case for transgenerational epigenetic inheritance in humans. Mamm. Genome.

[78] Milagro F.I., Mansego M.L., de Miguel C., Martinez J.A. (2013). Dietary factors, epigenetic modifications and obesity outcomes: Progresses and perspectives. Mol. Aspects Med..

